# Supplementary material for: Analysis of PPARγ Signaling Activity in Psoriasis
Source: Int J Mol Sci. 2021 Aug 10;22(16):8603. doi: 10.3390/ijms22168603 (PMC8395241; doi:10.3390/ijms22168603)
Supplement: Supplementary file 1 [file ijms-22-08603-s001.zip › Supplemental materials_Analysis of PPARg signaling activity in psoriasis/Pathway models/Models images and html files/Model of PPARG signaling in psoriasis/2696.html]

COL18A1


# Protein COL18A1

|  |  |
| --- | --- |
| URN | urn:agi-llid:1309 |
| Total Entities | 0 |
| Connectivity | 739 |
| Name | COL18A1 |
| Description | collagen type XVIII alpha 1 chain |
| Notes | This gene encodes the alpha chain of type XVIII collagen. This collagen is one of the multiplexins, extracellular matrix proteins that contain multiple triple-helix domains (collagenous domains) interrupted by non-collagenous domains. A long isoform of the protein has an N-terminal domain that is homologous to the extracellular part of frizzled receptors. Proteolytic processing at several endogenous cleavage sites in the C-terminal domain results in production of endostatin, a potent antiangiogenic protein that is able to inhibit angiogenesis and tumor growth. Mutations in this gene are associated with Knobloch syndrome. The main features of this syndrome involve retinal abnormalities, so type XVIII collagen may play an important role in retinal structure and in neural tube closure. Alternative splicing results in multiple transcript variants. [provided by RefSeq, Dec 2014] |
| Primary Cell Localization | Extracellular |

---

|  |  |
| --- | --- |
| Pathway | Proteins Involved in Diabetes Mellitus Type 1 |
|  | Proteins Involved in Ulcerative Colitis |
|  | Proteins Involved in Glioma |
|  | Proteins Involved in Endometriosis |
|  | Angiogenesis in Endometriosis |
|  | Proteins Involved in Age-Related Macular Degeneration |
|  | Proteins Involved in Cataract |
|  | VEGFA in Neovascular Glaucoma |
|  | FIGF -> AP-1 Expression Target |
|  | FIGF -> NCOR2 Expression Target |
|  | VEGF Signaling |
|  | TGFB1-ACVRL1 Expression Targets |
|  | VEGFA -> ATF/CREB/ELK-SRF Expression Targets |
|  | VEGFC -> CTNNB Expression Target |
|  | VEGFA -> NFATC Expression Targets |
|  | VEGFA -> STAT Expression Targets |
|  | VEGFC -> ATF Expression Target |
|  | VEGFA -> FOXO3A Expression Targets |
|  | VEGFA -> AP-1/CREBBP/MYC Expression Targets |
|  | VEGFA -> CTNNB/CTNND Expression Targets |
|  | VEGFA -> NCOR2 Expression Target |
|  | VEGFR -> CTNND Signaling |
|  | VEGFR -> CTNNB Signaling |
|  | VEGFR -> NFATC Signaling |
|  | VEGFR -> STAT Signaling |
|  | VEGFR -> AP-1/CREB/MYC Signaling |
|  | VEGFR -> FOXO3A Signaling |
|  | VEGFR -> ATF/CREB/ELK-SRF Signaling |
|  | Model of PPARG signaling in psoriasis |

---

|  |  |
| --- | --- |
| Group | Genes Associated with Mental Retardation and Brain Malformations in Epilepsy |

---

|  |  |
| --- | --- |
| MedScan ID | 1309 |

---

|  |  |
| --- | --- |
| LocusLink ID | 1309 |
|  | 80781 |
|  | 12822 |
|  | 85251 |

---

|  |  |
| --- | --- |
| Alias | collagen, type XVIII, alpha 1 |
|  | mEndo |
|  | OTTHUMP00000115473 |
|  | Col18a I |
|  | collagen XVIII |
|  | OTTHUMP00000115472 |
|  | FLJ34914 |
|  | KNO1 |
|  | collagen, type XVIII, alpha |
|  | alpha 1 type XVIII collagen isoform 1 |
|  | Knobloch syndrome protein |
|  | collagen type 18 |
|  | COL18A1 |
|  | OTTMUSP00000023766 |
|  | multi-functional protein mfp |
|  | Collagen alpha 1(XVIII) chain |
|  | murine endostatin |
|  | collagen alpha-1(XVIII) chain isoform 1 preproprotein |
|  | collagen, type XVIII, alpha I |
|  | MGC74745 |
|  | Knobloch syndrome gene |
|  | COL18A1\_2 |
|  | LOC51695 |
|  | procollagen, type XVIII, alpha |
|  | OTTMUSP00000023764 |
|  | human endostatin |
|  | procollagen, type XVIII, alpha I |
|  | recombinant human endostatin |
|  | FLJ27325 |
|  | alpha 1 type XVIII collagen |
|  | alpha 1 type XVIII collagen isoform 3 |
|  | rhEndostatin |
|  | type XVIII collagen |
|  | hEndo |
|  | procollagen, type XVIII, alpha 1 |
|  | alpha 1 type XVIII collagen isoform 2 |
|  | KS |
|  | KNO |
|  | antiangiogenic agent |
|  | endostatin |
|  | collagen alpha-1(XVIII) chain |
|  | alpha-1(XVIII) collagen |

---

|  |  |
| --- | --- |
| GO ID | 0005201 |
|  | 0030020 |
|  | 0046872 |
|  | 0001525 |
|  | 0009887 |
|  | 0007155 |
|  | 0001886 |
|  | 0030198 |
|  | 0008285 |
|  | 0030335 |
|  | 0008284 |
|  | 2000353 |
|  | 0042493 |
|  | 0051599 |
|  | 0007601 |
|  | 0005604 |
|  | 0005581 |
|  | 0062023 |
|  | 0005788 |
|  | 0070062 |
|  | 0031012 |
|  | 0005576 |
|  | 0005615 |
|  | 0030938 |

---

|  |  |
| --- | --- |
| KEGG ID | hsa:80781 |
|  | mmu:12822 |

---

|  |  |
| --- | --- |
| Organism | Homo sapiens {Organism urn:agi-taxid:9606} |
|  | Mus musculus {Organism urn:agi-taxid:10090} |
|  | Rattus norvegicus {Organism urn:agi-taxid:10116} |
|  | Homo sapiens |
|  | Mus musculus |
|  | Rattus norvegicus |

---

|  |  |
| --- | --- |
| Mouse chromosome position | 10 39.72 cM |

---

|  |  |
| --- | --- |
| OMIM ID | 120328 |
|  | 267750 |

---

|  |  |
| --- | --- |
| Rat chromosome position | 20p12 |

---

|  |  |
| --- | --- |
| Hugo ID | 2195 |

---

|  |  |
| --- | --- |
| Human chromosome position | 21q22.3 |

---

|  |  |
| --- | --- |
| Swiss-Prot Accession | D3DSM5 |
|  | P39060 |
|  | D3DSM4 |
|  | P39060.5 |
|  | P39061 |
|  | E9QPX1 |
|  | P39061.4 |
|  | F1LR02 |
|  | A8MVI4 |
|  | Q58EX6 |
|  | Q6RZ39 |
|  | Q6RZ40 |
|  | Q6RZ41 |
|  | Q8N4S4 |
|  | Q8WXI5 |
|  | Q96T70 |
|  | Q9UK38 |
|  | Q9Y6Q7 |
|  | Q9Y6Q8 |
|  | Q60672 |
|  | Q61434 |
|  | Q61437 |
|  | Q62001 |
|  | Q62002 |
|  | Q6NZK9 |
|  | Q6P1Y4 |
|  | Q8CCZ8 |
|  | Q9CRT2 |
|  | Q9JK63 |

---

|  |  |
| --- | --- |
| PIR ID | A56101 |
|  | B56101 |

---

|  |  |
| --- | --- |
| GenBank ID | NC\_000021 |
|  | NM\_130445 |
|  | NP\_569712 |
|  | NM\_130444 |
|  | NP\_569711 |
|  | NM\_030582 |
|  | NP\_085059 |
|  | NG\_011903 |
|  | AH013565 |
|  | AAR83296 |
|  | AAR83297 |
|  | AAR83298 |
|  | AL163302 |
|  | CAB90482 |
|  | BX322561 |
|  | BX322562 |
|  | BX322563 |
|  | CH471079 |
|  | EAX09338 |
|  | EAX09339 |
|  | EAX09340 |
|  | EAX09341 |
|  | EAX09342 |
|  | EAX09343 |
|  | EAX09344 |
|  | HF584235 |
|  | CCQ43732 |
|  | KF457341 |
|  | KF457343 |
|  | KF511425 |
|  | KX545276 |
|  | ANT47187 |
|  | AF018081 |
|  | AAC39658 |
|  | AF018082 |
|  | AAC39659 |
|  | AF184060 |
|  | AAF01310 |
|  | AF333246 |
|  | AF333247 |
|  | AAK50626 |
|  | AF416592 |
|  | AAL37720 |
|  | AK098216 |
|  | AK130835 |
|  | BC033715 |
|  | AAH33715 |
|  | BC063833 |
|  | AAH63833 |
|  | CN389577 |
|  | L22548 |
|  | AAA51864 |
|  | P39060 |
|  | NC\_000076 |
|  | XM\_006513174 |
|  | XP\_006513237 |
|  | XM\_006513173 |
|  | XP\_006513236 |
|  | XM\_006513176 |
|  | XP\_006513239 |
|  | XM\_011243346 |
|  | XP\_011241648 |
|  | NM\_009929 |
|  | NP\_034059 |
|  | NM\_001109991 |
|  | NP\_001103461 |
|  | XM\_011243347 |
|  | XP\_011241649 |
|  | AC055777 |
|  | AC159334 |
|  | AH006757 |
|  | AAC52901 |
|  | AAC52902 |
|  | AAC52903 |
|  | CH466553 |
|  | EDL31819 |
|  | EDL31820 |
|  | AF257775 |
|  | AAF69009 |
|  | AK014292 |
|  | BAB29249 |
|  | AK031798 |
|  | BAC27554 |
|  | BC008227 |
|  | BC043697 |
|  | BC062931 |
|  | BC064817 |
|  | AAH64817 |
|  | BC066080 |
|  | AAH66080 |
|  | CF171159 |
|  | D17546 |
|  | BAA04483 |
|  | L16898 |
|  | AAA37434 |
|  | L22545 |
|  | AAA19787 |
|  | U03714 |
|  | AAA20657 |
|  | U11636 |
|  | AAC52178 |
|  | U11637 |
|  | AAC52179 |
|  | P39061 |
|  | NC\_005119 |
|  | NM\_053489 |
|  | NP\_445941 |
|  | AC\_000088 |
|  | AABR07044596 |
|  | AAHX01100117 |
|  | AAHX01100118 |
|  | AAHX01100119 |
|  | AAHX01100120 |
|  | AAHX01100121 |
|  | AAHX01100122 |
|  | AAHX01100123 |
|  | AAHX01100124 |
|  | AAHX01100125 |
|  | AAHX01100126 |
|  | AAHX01100127 |
|  | CH473988 |
|  | EDL97114 |
|  | EDL97116 |
|  | AF189709 |
|  | AAF00975 |
|  | AJ236873 |
|  | CAB44263 |
|  | CA505156 |
|  | CA510744 |
|  | CA513367 |
|  | CO385937 |
|  | DV216514 |
|  | FM035839 |
|  | FM037592 |
|  | FQ223217 |

---

|  |  |
| --- | --- |
| Swiss-Prot ID | COIA1\_HUMAN |
|  | COIA1\_MOUSE |

---

|  |  |
| --- | --- |
| Cell Localization | Secreted |
|  | Extracellular space |
|  | Extracellular matrix |
|  | Basement membrane |

---

|  |  |
| --- | --- |
| Ensembl ID | ENSG00000182871 |
|  | ENSP00000347665.5 |
|  | ENST00000355480.10 |
|  | ENSP00000352798.4 |
|  | ENST00000359759.8 |
|  | ENSP00000383191.2 |
|  | ENST00000400337.6 |
|  | ENSMUSG00000001435 |
|  | ENSMUSP00000101049.1 |
|  | ENSMUST00000105409.7 |
|  | ENSMUSP00000080358.6 |
|  | ENSMUST00000081654.12 |
|  | ENSMUSP00000072538.4 |
|  | ENSMUST00000072755.11 |
|  | ENSRNOG00000001229 |
|  | ENSRNOP00000047962.4 |
|  | ENSRNOT00000045315.4 |

---

|  |  |
| --- | --- |
| MGI ID | MGI:88451 |

---

|  |  |
| --- | --- |
| RGD ID | 70936 |

---
